# Obesity Promotes Alterations in Iron Recycling

**DOI:** 10.3390/nu7010335

**Published:** 2015-01-06

**Authors:** Marta Citelli, Thaís Fonte-Faria, Vany Nascimento-Silva, Mariana Renovato-Martins, Raphael Silva, Aderval Severino Luna, Simone Vargas da Silva, Christina Barja-Fidalgo

**Affiliations:** 1 Departamento de Nutrição Básica e Experimental, Instituto de Nutrição, Universidade do Estado do Rio de Janeiro, Rio de Janeiro, RJ 21941-590, Brazil; E-Mail: raphasilva02@gmail.com; 2 Instituto de Biologia Roberto Alcantara Gomes, Universidade do Estado do Rio de Janeiro, Rio de Janeiro, RJ 20551-030, Brazil; E-Mails: thaisfaria09@gmail.com (T.F.-F.); vanysilva@hotmail.com (V.N.-S.); m_renovatomartins@yahoo.com.br (M.R.-M.); si_vargas@oi.com.br (S.V.-S.); barja-fidalgo@uerj.br (C.B.-F.); 3 Instituto de Química, Universidade do Estado do Rio de Janeiro, Rio de Janeiro, RJ 20551-030, Brazil; E-Mail: asluna@uerj.br

**Keywords:** hepcidin, ferroportin, ER stress, obesity, iron, bioavailability

## Abstract

Hepcidin is a key hormone that induces the degradation of ferroportin (FPN), a protein that exports iron from reticuloendothelial macrophages and enterocytes. The aim of the present study was to experimentally evaluate if the obesity induced by a high-fat diet (HFD) modifies the expression of FPN in macrophages and enterocytes, thus altering the iron bioavailability. In order to directly examine changes associated with iron metabolism *in vivo*, C57BL/6J mice were fed either a control or a HFD. Serum leptin levels were evaluated. The hepcidin, divalent metal transporter-1 (DMT1), FPN and ferritin genes were analyzed by real-time polymerase chain reaction. The amount of iron present in both the liver and spleen was determined by flame atomic absorption spectrometry. Ferroportin localization within reticuloendothelial macrophages was observed by immunofluorescence microscopy. Obese animals were found to exhibit increased hepcidin gene expression, while iron accumulated in the spleen and liver. They also exhibited changes in the sublocation of splenic cellular FPN and a reduction in the FPN expression in the liver and the spleen, while no changes were observed in enterocytes. Possible explanations for the increased hepcidin expression observed in HFD animals may include: increased leptin levels, the liver iron accumulation or endoplasmic reticulum (ER) stress. Together, the results indicated that obesity promotes changes in iron bioavailability, since it altered the iron recycling function.

## 1. Introduction

The liver is the main organ responsible for endocrine regulation of iron homeostasis and dictates the amount that is stored; whereas, the homeostatic system fine tunes the availability of iron in the plasma, which, in turn, supplies iron to cells and tissues, thus safeguarding against iron excess. The liver responds to erythropoietic demand and channels most of the systemically-available iron toward synthesis of heme. The liver orchestrates the flow of iron, by regulating the synthesis of hepcidin, a hormone that controls the availability of iron through its interaction with ferroportin (FPN) [1]. Ferroportin is a transmembrane protein responsible for exporting iron from cells, such as duodenal enterocytes and macrophages. The action of hepcidin on FPN promotes its internalization and subsequent degradation [1]. Thus, hepcidin prevents the transfer of iron from enterocytes to the portal circulation and disrupts the ability of reticuloendothelial macrophages to furnish iron to the bloodstream.

Several studies have indicated that weight or body composition can be contributing factors leading to iron deficiency. Data from NHANES III (National Health and Nutrition Examination Survey) indicated that iron deficiency in overweight American children was two-times more prevalent than in children of normal weight [2], and similar results were also reported in the adult population [3]. These observations led to the hypothesis that these two morbidities are associated [4,5,6], which would indicate that the nutritional management for obese individuals should also consider the investigation and/or treatment of iron deficiency. During the last few years, iron stores have been linked to an increased risk of metabolic complications, such as type 2 diabetes. Therefore, the potential therapeutic role of iron depletion therapy for the prevention of clinical complications is beginning to be evaluated in controlled trials [7].

Additionally, intracellular endoplasmic reticulum (ER) stress induced by nutrient excess has been shown to stimulate hepcidin expression and leads to hypoferremia in mice [8,9]. A parallel finding, indicated that leptin, an adipokine that plays an important role in the regulation of satiety [10], has the ability to activate hepcidin signaling through the JAK/STAT (Janus kinase/signal transduction and activators of transcription) pathway in human hepatoma cells (Huh7) [11].

Therefore, obesity, induced by a high-fat diet, can lead to ER stress [12,13,14] or promote leptin secretion, and both intersect, leading to a common outcome, the induction of hepcidin transcription. Although there is a common sense that the increased levels of hepcidin found in obesity exert an effect on the expression of FPN present in the duodenum and reticulum endothelial macrophages [6], leading to reduced iron bioavailability, this hypothesis is experimentally explored here for the first time.

## 2. Experimental Section

### 2.1. Materials

The materials used in this study were obtained from various sources. Pronase was procured from Sigma (St. Louis, MO, USA). TRIzol reagent was obtained from Invitrogen (Canada), while DNase was purchased from Promega Corporation (Madison, WI, USA). Primers used for RT-PCR were generated by Integrated DNA Technologies, Inc. (Coralville, IA, USA). The High Capacity cDNA Reverse Transcription Kit was purchased from Applied Biosystems (Foster City, CA, USA). The Rotor-Gene SYBR Green PCR Kit was obtained from Qiagen (Hilden, Germany). The PVDF membranes (Hybond-P) and rainbow markers were procured from Amersham Biosciences (Buckinghamshire, UK), and streptavidin was from Caltag Laboratories. The primary anti-ferroportin antibody was purchased from Abcam. The biotin-conjugated secondary antibody was obtained from Santa Cruz Biotechnology, (Santa Cruz, CA, USA).

### 2.2. Ethics Statement

This study was carried out in strict accordance with the recommendations in the Guide for the Care and Use of Laboratory Animals of the National Institutes of Health. The protocol was approved by the Committee on the Ethics of Animal Experiments at the Instituto de Biologia Roberto Alcantara Gomes of the Universidade do Estado do Rio de Janeiro (Permit Number CEA/047/2009). All surgery was performed under ketamine and xylazine anesthesia, and all efforts were made to minimize suffering.

### 2.3. Animals and Diet Protocol

C57BL/6J male mice were obtained from animal facilities maintained by the Instituto Nacional do Câncer (Rio de Janeiro, Brazil) and housed under controlled room temperature (25 °C ± 1 °C) and 60% humidity with an artificial dark-light cycle (light from 7:00 a.m. to 7:00 p.m.). Mice were randomly divided into two groups subjected to different feeding regimens: standard chow for rodents (Control group—CONT, 3.8 kcal/g of chow) or high-fat diet chow (High Fat Diet group—HFD, 4.9 kcal/g of chow calorically-enhanced by hydrogenated vegetable fat). Upon weaning, the animals were fed these diets for 30 weeks. The constitution of the standard chow was: 14% energy value obtained from protein, 10% from fat and 76% from carbohydrate; and the HFD derived 14% of the energy value from protein, 54% from fat and 32% from carbohydrates. Further information about the diet composition determination can be found in Table S1. Both diets included a micronutrient mineral mix, supplemented according to the recommendations of the American Institute of Nutrition (AIN-93G) and based on a previous publication [15]. The body weight of each animal was evaluated on each experimental day.

### 2.4. Intraperitoneal Glucose Tolerance Test

Prior to the glucose tolerance test, animals were fasted overnight for 10 ± 2 h and wrapped in a towel the following morning to minimize stress. The baseline blood glucose measurements were performed using a drop of tail blood. Subsequently, the animals received a glucose challenge (1 g/kg of body weight, i.p.) followed by repeated sampling of blood glucose readings at 30, 60 and 120 min. The glucose measurements were taken using a handheld glucometer (Accu-Chek Roche^®^; Indianapolis, IN, USA) [16].

### 2.5. Sample Collection

After completion of each experiment, mice were euthanized by withdrawing blood from the heart under anesthesia, mixing ketamine (50 mg/kg) with xylazine (20 mg/kg). A small piece of small intestine, closest to the stomach (4 cm), the spleen and the liver were collected and transferred to tubes, snap frozen in liquid nitrogen and stored at −80 °C until RNA extraction, western blotting and atomic absorption spectrometry (AAS) analysis.

### 2.6. Measurements of Leptin and IL-6

Serum levels of leptin and IL-6 were measured using appropriate ELISA kits (Peprotech, Rocky Hill, NJ, USA; and Cayman Chemical, Ann Arbor, MI, USA; respectively), following the manufacturer’s instructions.

### 2.7. RNA Extraction and qRT-PCR Analysis

Total RNA was obtained from tissues and macrophages and extracted using TRIzol reagent, followed by DNase digestion. The cDNA was generated using 1 μg RNA and the High Capacity cDNA Reverse Transcription Kit (Applied Biosystems, Foster City, CA, USA). Gene-specific primers were used for the real-time analysis and are shown in Table 1. Mus musculus TNF-alpha (NM_013693) primers were purchased from Qiagen (Mm_Tnf_1_SG QuantiTect Primer Assay). The remaining primers (Table 1) were selected using the Primer Express Software (Applied Biosystems, Foster City, CA, USA) or Primer3 Software and purchased from Integrated DNA Technologies, Inc. (Coralville, IA, USA). Samples were run as triplicates, and the PCR reaction consisted of 40 cycles using the Rotor Gene SYBR Green PCR Kit and processed in Rotor Gene (Qiagen). The analysis of RT-PCR output data followed the manufacturer-suggested ΔCt method. Cycle thresholds (Ct) were measured, and the relative expression of genes was calculated by comparison of Ct values, using one calibrating sample from the control group. All samples were normalized to the housekeeping gene, glyceraldehydes-3-phosphate dehydrogenase. Melt-curve analysis was used to confirm the production of a single amplicon for each gene tested.

**Table 1:** List of primers used for quantitative real-time PCR and XBP1 splicing analysis.

Target Gene	Forward Primer (5′–3′)	Reverse Primer (5′–3′)	GenBank Accession Number
DMT1	CTCCACCATGACTGGAACCT	TTCAGGAATCCCTCCATGAC	NM_001146161
Ferritin	TGATGAAGCTGCAGAACCAG	GTGCACACTCCATTGCATTC	NM_010239
Ferroportin	TTGCAGGAGTCATTGCTGCTA	TGGAGTTCTGCACACCATTGAT	NM_016917
GAPDH	CCTCGTCCCGTAGACAAAATG	TGAAGGGGTCGTTGATGGC	NM_008084
Hepcidin	CCTATCTCCATCAACAGATG	AACAGATACCACACTGGGAA	NM_032541
XBP1	GAACCAGGAGTTAAGAACACG	AGGCAACAGTGTCAGAGTCC	NM_0012717301

### 2.8. Semi-Quantitative RT-PCR

XBP1 (X-box binding protein) mRNA spliced forms were analyzed by semiquantitative RT-PCR using cDNA obtained as specified in the previous section from mouse livers (*n* = 8/group). The PCR products were run on a 2.5% agarose gel, and the primers utilized were previously reported by Vecchi *et al.* [2] (Table 1).

### 2.9. Western Blotting

The total protein content of cell extracts was determined by Bradford’s method [17]. Samples were resolved to SDS-PAGE, and proteins were transferred to PVDF membranes. Rainbow markers were run in parallel to estimate molecular weights. Membranes were blocked with Tween-PBS (0.1% Tween-20) containing 5% bovine serum albumin and incubated with specific primary antibodies: anti-ferroportin (1:1000) (Abcam; ~65 KDa); anti-eIF2-alpha (1:1000) (Cell Signaling; ~40 KDa); anti-tubulin (1:500) (Santa Cruz Biotechnology; ~55 KDa); anti-actin (1:1000) (Cell Signaling; ~42 KDa). After extensive washing in Tween-PBS, PVDF sheets were incubated with the appropriate secondary biotin-conjugated antibody (1:10,000) (Santa Cruz Biotechnology, Santa Cruz, CA, USA) for 1 h and then incubated with horseradish peroxidase-conjugated streptavidin (1:10,000). Immunoreactive proteins were visualized using the ECL system. Membranes were stripped with stripping buffer (62.5 mM Tris-HCl, 2% SDS and 100 mM b-mercaptoethanol) and re-probed similarly with anti-actin or anti-tubulin antibody. Films were scanned and semi-quantitatively analyzed. The cellular extracts were normalized to actin or tubulin, while the bands were quantified by densitometry, using ImageJ 1.34 s Software (NIH, Bethesda, MD, USA).

### 2.10. Isolation and Culture of Splenic Macrophages

We utilized the standard pronase digestion technique previously described using human Kupffer cells [18]. Briefly, the spleens (*n* = 6/group) were excised and minced before incubation with Grey’s balanced salt solution (GBSS)-pronase solution, while being continuously stirred at 37 °C for 60 min. DNase (0.8 g/mL) was added to prevent the cells from clumping. The spleen slurry was filtered through gauze mesh, washed with culture media and centrifuged two times at 600× *g* for 5 min. Cells were then resuspended in PBS with DNase (0.8 g/mL). Purified nonparenchymal cells were washed and cultured in RPMI medium supplemented with 100,000 U/L penicillin, 100 mg/L streptomycin and 15 mM HEPES. Reticuloendothelial macrophages were enriched by differential adherence to tissue culture plates. Cells (2.0 × 10^6^ cells/well in a 24-well plate) were plated in culture plates and incubated at 37 °C for 3 h before washing and overnight incubation in culture media containing 5% fetal bovine serum (FBS). All experiments were subsequently performed after the cells were washed three times with serum-free media. For each experiment, the cells were isolated from a single spleen.

### 2.11. Immunofluorescence Microscopy

#### Immunocytochemistry

Macrophages were isolated as described above and incubated for 3 h at 37 °C and 5% CO_2_ during adhesion onto glass coverslips in 24-well plates. Immunofluorescence studies were performed as previously described [19]. Briefly, the cells were fixed with 4% paraformaldehyde/4% sucrose in PBS for 20 min, permeabilized in PBS/0.2% Triton X-100 for 1 h at room temperature and blocked with 5% BSA in PBS for 30 min. The cells were then incubated overnight with polyclonal anti-ferroportin Ab (1:400 dilution; Abcam, AB58695) at 4 °C. Subsequently, the cells were washed three times with PBS and incubated with secondary biotin-conjugated anti-rabbit IgG (1:400 dilution) followed by incubation with Alexa Fluor 555 (Invitrogen, Paisley, UK) for 1 h at room temperature. Samples were then mounted using ProLong Gold antifade reagent coupled with 4,6-diamidino-2-phenylindole (DAPI) for nuclear staining (Invitrogen, Paisley, UK). Acquisition was performed using an Olympus BX40 microscope equipped for epifluorescence using a 40× objective.

### 2.12. Iron Concentrations in Liver and Spleen

Liver and spleen iron concentrations were measured by flame atomic absorption spectrometry (Perkin-Elmer AA300, USA) following the dry ashing procedure. Ashes were further re-suspended in HNO_3_ 0.1 mol/L solution. The liver and spleen iron concentrations were expressed as the iron content within 1 g of ash.

### 2.13. Statistical Analysis

The data were expressed as the mean ± standard error and analyzed by the two-tailed unpaired Student’s *t*-test or analysis of variance (ANOVA). When appropriate, individual comparisons were subsequently tested using the Tukey *t*-test for unpaired values. Differences were considered statistically significant when *p* < 0.05. The data were analyzed using GraphPad Prism version 5.00 for Windows (GraphPad Software, La Jolla, CA, USA).

## 3. Results

### 3.1. Effects of High-Fat Diet on Metabolic Parameters

**Figure 1:**
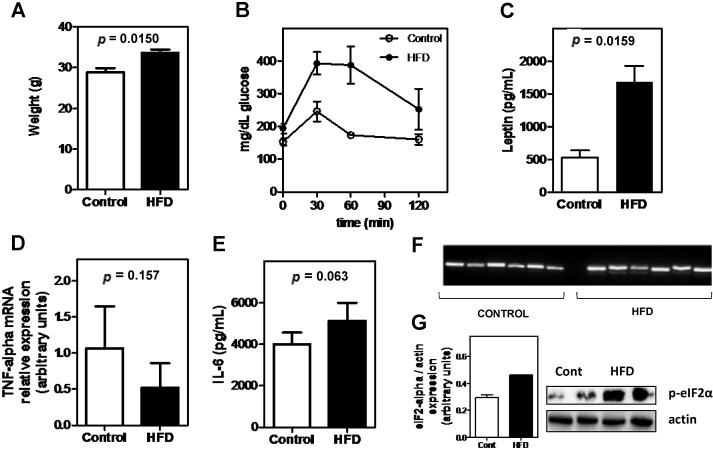
Effect of a high-fat diet (HFD): (**A**) on body weight; (**B**) on glucose tolerance; (**C**) on leptin serum levels; (**D**) on liver TNF-alpha mRNA expression; (**E**) on IL-6 serum levels; and ER (endoplasmic reticulum) stress, as demonstrated by (**F**) the differential splicing of the XBP-1 (X-box protein) gene (**G**) or the phosphorylation of eIF2-alpha. Data represent the mean ± SE (*n* = 4–8/group).

Animals subjected to the highly caloric, fatty diet had increased body weight, accompanied by lower glucose tolerance and elevated leptin levels by the end of the study (Figure 1A–C). Considering that obesity is implicated in eliciting ER stress and that inflammatory parameters were not overtly increased (Figure 1D,E), we assessed whether the hepatic tissue was undergoing ER stress by verifying whether differential splicing occurred at the XBP-1 gene or eIF2-alpha was being activated by phosphorylation. As observed in Figure 1F,G, the HFD was able to induce ER stress.

### 3.2. Obesity Altered the Bioavailability of Hepatic and Splenic Iron

**Figure 2:**
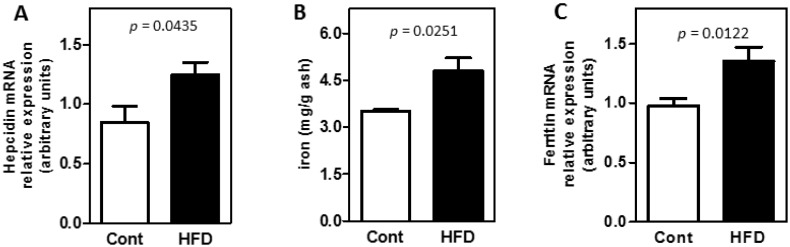

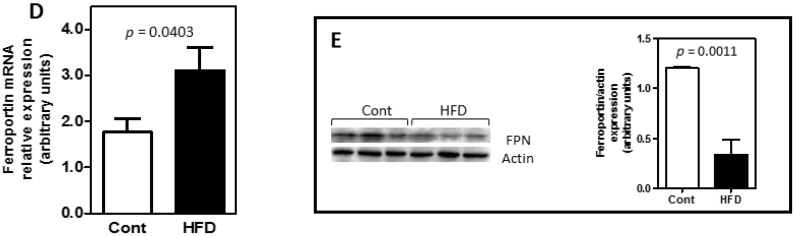
Obesity induced hepatic changes. The high-fat diet (HFD) induced: (**A**) hepcidin mRNA expression; iron accumulation; as shown by (**B**) the amount of iron, (**C**) ferritin and (**D**) ferroportin mRNA expression. (**E**) FPN protein expression analysis was done using liver total extracts. The white bars represent the control group, and the black bars represent the HFD group. Data represent the mean ± SE (*n* = 5–8/group).

Obesity modulated the transcription of some genes involved with the regulation of iron bioavailability. Obese animals had increased hepatic hepcidin mRNA levels (Figure 2A) and exhibited iron accumulation (Figure 2B). This outcome was accompanied by transcriptional induction of ferritin and FPN (Figure 2C,D) and by the reduction on FPN protein expression (Figure 2E).

**Figure 3:**
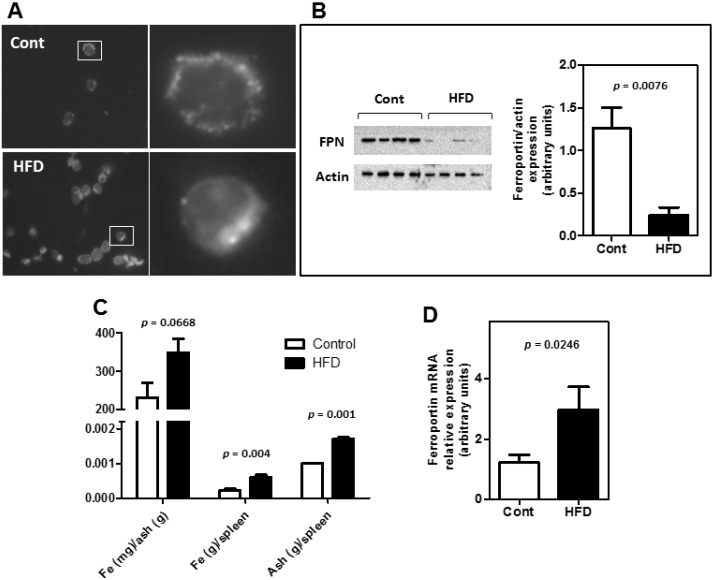
Administration of a high-fat diet (HFD) induced obesity and promoted altered iron homeostasis in the spleen. (**A**,**B**) In reticuloendothelial macrophages obtained from obese mice, ferroportin presented different sublocation and reduced expression. (**C**) Splenic iron retention was evaluated by quantifying iron content by flame atomic absorption spectrometry. (**D**) Ferroportin mRNA expression was evaluated by real-time PCR. The white bars represent the control group, and the black bars represent the HFD group. Data represent the mean ± SE (*n* = 4–8/group).

One of the most prominent functions of the spleen is its ability to recycle iron due to macrophage phagocytosis of senescent red blood cells. Immunocytochemistry microscopy revealed that the FPN protein was distributed differently between groups of splenic reticuloendothelial macrophages (Figure 3A). High levels of FPN were localized at the cell membranes of control animals, while the HFD group exhibited only a sparse, scattered distribution of this protein at the cell membrane. Splenic reticuloendothelial macrophages from obese animals presented reduced FPN expression (Figure 3B). In parallel, obesity caused enhanced levels of iron in the spleen (Figure 3C) and increased FPN mRNA expression in splenic macrophages (Figure 3D).

### 3.3. The Expression of Key Molecules Involved with Intestinal Iron Absorption Was Not Altered by Obesity

**Figure 4:**
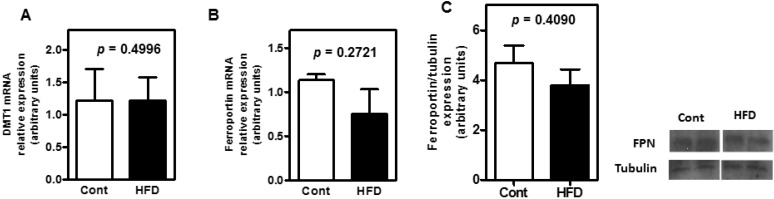
Effect of HFD on mRNA levels of the (**A**) duodenum divalent metal transporter 1 (DMT1) and (**B**) ferroportin (FPN). Relative mRNA levels were analyzed by real-time RT-PCR. Results are expressed as a relative comparison to the control condition. Each value represented the means ± SE (*n* = 8/group). (**C**) Ferroportin expression in the duodenum of obese animals was assessed by immunoblotting analysis.

To investigate whether diet-induced obesity was able to modulate the expression of genes encoding key proteins involved in duodenal iron absorption, we evaluated divalent metal transporter 1 (DMT1) and FPN mRNA expression levels in C57BL/6J mice. As demonstrated in Figure 4, no significant differences were found in the transcript levels of these genes between control and HFD groups. Moreover, these data are in agreement with the analysis of protein expression.

## 4. Discussion

Bioavailability of iron can be modified either due to absorptive causes or due to nutritional causes, such as vitamin A deficiency [20,21]. The knowledge that iron deficiency is present at higher levels in the obese population was derived from associative studies and led us to the hypothesis that iron metabolism would be modified in these individuals. For this reason, we analyzed the potential of obesity to modulate molecular mechanisms that are instrumental in facilitating iron bioavailability.

The animals that were submitted to HFD exhibited excess weight, reduced glucose tolerance and increased leptin levels. Besides regulating food intake and energy expenditure, leptin regulates the expression of the iron regulatory hormone, hepcidin, as demonstrated in human hepatoma cells [11]. These results agree with the idea that increased production of leptin in overweight individuals may substantially contribute to the observed aberrant iron status in this population and are consistent with the results found in the present study, which showed increased leptin secretion and hepcidin mRNA expression in obese animals.

In addition to the leptin effect, ER stress is also known to induce hepcidin transcription, as was elegantly demonstrated by Vecchi *et al.* [8] and Oliveira *et al.* [9]. This finding furthered the understanding that iron homeostasis and obesity may be closely related. Additional studies have shown that excessive, chronic nutrient intake causes ER stress in the adipose tissue of ob/ob mice and mice fed high-fat diets [12,13,14]. The link between ER stress and metabolism was shown in a study where mice deficient in Xbox-binding protein-1 (XBP-1), a transcription factor that modulates the ER stress response, develop insulin resistance. This demonstrates that ER stress is a central feature underlying peripheral insulin resistance [22]. It is likely, therefore, that ER stress was regulated by inflammatory responses. However, Pierre *et al.* [23] refuted this hypothesis, demonstrating that Toll-like receptor 4 (TLR4) signaling does not mediate lipid-induced ER stress via activation of the NF-κB pathway. Here, analysis of cytokine mRNA levels regulated by NF-κB, namely TNF-α and IL-6, remained unchanged after administration of HFD to mice, while they also presented ER stress (Figure 1F,G). Although there are consistent data indicating that HFD increases leptin levels and causes ER stress, more studies are necessary to determine whether these factors are responsible for the increased hepcidin expression observed in HFD-fed animals.

In order to better understand the molecular mechanisms involved in the hepcidin mRNA induction observed in the obese animals, we investigated the mRNA expression of hemochromatosis gene (Hfe), bone morphogenetic proteins 2 and 4 (BMP2, BMP4) and hemojuvelin (Hjv), because these molecules are known to be involved in hepcidin signaling. We observed no differences in their expression between groups (Figure S1, Table S2).

The expected consequence of increased hepcidin expression is the reduction of iron bioavailability, mediated by changes in FPN phosphorylation, internalization and lysosomal degradation in enterocytes and reticuloendothelial macrophages. An interesting outcome of this study, regarding obesity, was the finding that FPN localization within splenic reticuloendothelial macrophages was altered (Figure 3A). This change in FPN localization was accompanied by diminished FPN protein expression (Figure 3B), by iron trapping in the spleen (Figure 3C) and increased FPN transcription (Figure 3D). As demonstrated earlier, the amount of macrophage FPN mRNA was found to correlate with the concentration of intracellular iron, which varies substantially with cellular iron status and increased iron loading [24,25]. Studies that employed the transcriptional inhibitor, actinomycin D, provided additional evidence that macrophage FPN is regulated transcriptionally by iron [25], and this finding corroborates the increased iron concentration observed here.

The intestinal epithelium is another site at which hepcidin exerts its action. Nevertheless, the duodenal FPN protein expression was not modified by obesity, and similarly, DMT1 and FPN mRNA levels remained unaltered in this tissue (Figure 4). Chaston *et al.* [26] showed that a cell-type-specific response exists. When hepcidin levels are increased, the primary targets of hepcidin are the iron-recycling macrophages present in the spleen, rather than enterocytes. In agreement with these findings, we showed that obesity induced hepcidin gene expression in the liver. This increase in hepcidin was found to negatively impact iron recycling without affecting FPN expression within enterocytes or the transcription of key genes that regulate iron duodenal absorption. The iron absorption was not evaluated in the present study. Nevertheless, consistent with our results, Aeberli *et al.* [5] observed that overweight children presented iron bioavailability comparable with normal weight children. Some studies have suggested that adiposity predicts decreased intestinal iron absorption [27,28]. However, the contribution of body mass index (BMI) to the reduction in the iron absorption seemed to be low (*r*^2^ = 0.051; *p* = 0.030) [27]. Thus, the BMI contribution to the reduction in the iron absorption possible may depend on the inflammation severity. Additionally, while it is now widely recognized that inflammation affects commonly-used markers of iron status, there is still a lack of consensus on how to best adjust for these effects [29].

It was found, also in other studies, that hepcidin expression was increased in mice subjected to an HFD (60% increase in caloric value), although iron did not accumulate in the liver [30]. Le Guenno *et al.* [31] observed a reduction in the hepcidin mRNA expression of rats submitted to an HFD while iron was diminished in the tissues. Recently, Orr *et al.* [32] also observed diminished iron levels in tissues. Sonnweber *et al.* [33] observed that animals fed an HFD had reduced iron absorption via a hepcidin-independent mechanism, although there were no differences in the iron tissue content of these animals. Ahmed and Oates [34] observed that the amount of dietary fat affected tissue iron levels, however, not the transcription of hepcidin in rats.

On the other hand, dysmetabolic iron overload syndrome (DIOS) is detected in about one third of patients with nonalcoholic fatty liver disease and is characterized by hepatic iron overload associated with insulin resistance features [7]. Our results show similar characteristics to the DIOS. In the present model, liver hepcidin expression and serum leptin were enhanced in the HFD group, and these mice also demonstrated insulin resistance.

Possible explanations for the discrepancies found among the studies may be related to the length of time the present animals were fed the high-fat diet. Here, animals were fed the HFD for a longer time, which may have led to accumulation of iron within tissues over time. Additionally, this is supported by the evidence that hepcidin levels are increased with the maintenance of the ER stress response [9].

## 5. Conclusions

**Figure 5:**
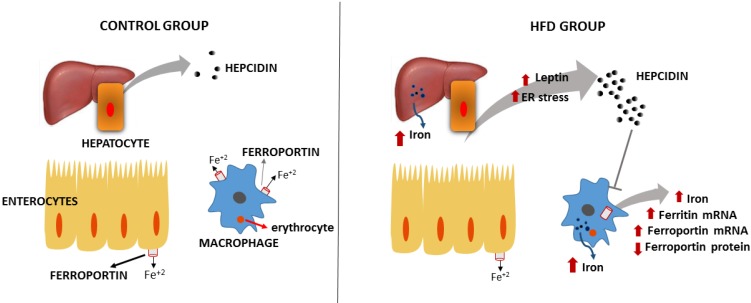
Representative model of the iron metabolism of high-fat diet (HFD)-fed animals. Iron recycling is altered by the HFD. The increased secretion of leptin, iron accumulation and the endoplasmic reticulum (ER) stress represent potent inducers of hepcidin synthesis, which may further change the sublocation of splenic reticuloendothelial ferroportin. Iron accumulates in the spleen, which may induce the ferritin and ferroportin mRNA expression. However, duodenal divalent metal transporter 1 (DMT1) and ferroportin (FPN) mRNA expression are not induced by HFD, and no changes in total duodenal FPN expression are found.

Finally, when considering the complex role of obesity on iron homeostasis, we conclude from the present results that obesity alters some of the molecular mechanisms involved in iron metabolism, thereby reducing its bioavailability, as represented in Figure 5.

## Supplementary Files

Supplementary File 1
