# Supplementary material for: Obesity Promotes Alterations in Iron Recycling
Source: Nutrients. 2015 Jan 6;7(1):335–48. doi: 10.3390/nu7010335 (PMC4303843; doi:10.3390/nu7010335)
Supplement: Supplementary File 1 [file nutrients-07-00335-s001.docx]

Supplementary Information

**Table S1.** Proximate composition of the diets.

| **Component** | **Control (X ± SD)** | **HFD (X ± SD)** |
| --- | --- | --- |
| Lipid (g/100g) | 4.5 (0.1) | 29.7 (0.5) |
| Carbohydrate (g/100g) | 71.0 (0.6) | 39.8 (2.3) |
| Protein (g/100g) | 12.9 (1.1) | 17.1 (1.9) |
| Ashes (g/100g) | 4.7 (0.2) | 5.3 (0.3) |
| Moisture (g/100g) | 7.1 (1.8) | 8.0 (0.6) |
| Lipid (% Kcal) | 10.7 ( 0.4) | 54.0 (0.3) |
| Carbohydrate (% Kcal) | 75.6 (0.6) | 32.2 (1.5) |
| Protein (% Kcal) | 13.7 (0.9) | 13.9 (1.7) |
| Kcal/g | 3.8 (0.1) | 4.9 (0.1) |

**Figure S1.** BMP2, BMP6, HFE and HJV mRNA expression. Effect of a high fat diet (HFD) on (**A**) liver Bone Morphogenetic Protein 2 (BMP2), (**B**) Bone Morphogenetic Protein 6 (BMP6), (**C**) HFE and (**D**) (Hemojuvelin) HJV mRNA expression. Data represents mean ± SE
(*n* = 4–7/group).

**Table S2.** List of primers used for quantitative real time PCR analysis.

| **Target gene** | **Forward primer (5′–3′)** | **Reverse primer (5′–3′)** |
| --- | --- | --- |
| BMP6  BMP2  HFE  HJV | AACCTTTCTTATCAGCATTTACCA  ATGGTGGCCGGGACCCGCTGTCTTG  TGTGAGGTGCATGAAGACAACAG  CCAGGCTGAGGTGGACAATC | GTGTCCAACAAAAATAGGTCAGAG  ACGACACCCGCAGCCCTCCACAAC  TCTTGCCCGTCATAACCATATCT  GTCGGTCGCCCCCATT |

Methods

Proximate Analyses

Analyzes were made by homogenizing 100 g of each diet, produced in two different batches
(Table S1).

1. Moisture—The moisture content was measured using an air-oven and following official methods of the Association of Official Analytical Chemists [1]. The samples were dried at 105^o^C until they achieved a constant weight, whereas the percentage of moisture was obtained with the equation below:

| % moisture = 1 − [(weight dry sample) − (weight wet sample)] × 100 | (1) |
| --- | --- |

2. Lipid—Determination of the lipid content was performed following the Sohxlet method previously described [1]. Petroleum ether was used for the extraction, whereas the percentage of lipid was obtained with the equation below:

| % lipid = [(weight of extraction cup + residue) – (weight of extraction cup)] ×  100/weight sample | (2) |
| --- | --- |

3. Protein—The total amount of nitrogen in the sample was determined according to the described method. A nitrogen-to-protein conversion factor of 6.25 was used for the determination of the protein present in the samples.

4. Ash Content—A dry ashing method was used to determine the ash content [1]. The samples were incinerated in a furnace (Furnace 62700, Barnstead/Thermolyne, Dubuque, IA, USA) at 550°C. An ash solution was prepared by dissolving the ash in 100 mL of 1 M HCl.

5. Carbohydrate—The total carbohydrate content (%) of the samples was calculated by the difference method [1].

RNA Extraction and qRT-PCR Analysis

Total RNA was obtained from tissues liver using Trizol reagent, followed by DNase digestion. The cDNA was generated using 1 μg RNA and the High Capacity cDNA Reverse Transcription Kit (Applied Biosystems, Foster City, CA, USA). Gene-specific primers were used for the real-time analysis and are shown in Table S2. The primers were purchased from Exxtend, Inc. (São Paulo, Brazil). Samples were run as triplicates and the PCR reaction consisted of 40 cycles using the Rotor Gene SYBR Green PCR kit (Qiagen, Valencia, CA, USA), and processed in Rotor Gene (Corbett Research, Sydney, Australia). The analysis of RT-PCR output data followed the manufacturer-suggested ΔCt method. Cycle thresholds (Ct) were measured and the relative expression of genes was calculated by comparison of Ct values. All samples were normalized to the housekeeping gene glyceraldehydes-3-phosphate dehydrogenase. Melt-curve analysis was used to confirm the production of a single amplicon for each gene tested.

Reference

1. Association of Official Analytical Chemistry—AOAC Internacional (2002) Official Methods of Analysis of Association of Official Analytical Chemist International. Available online: http://www.
aoac.org/iMIS15_Prod/AOAC_Member/PUBSCF/OMACF/OMAP_M.aspx?&WebsiteKey=2e25ab5a-1f6d-4d78-a498-19b9763d11b4&hkey=5142c478-ab50-4856-8939-a7a491756f48&CCO=8 (accessed on 27 November 2014).

© 2015 by the authors; licensee MDPI, Basel, Switzerland. This article is an open access article distributed under the terms and conditions of the Creative Commons Attribution license (http://creativecommons.org/licenses/by/4.0/).
